# Preparing for the future of public health: ecological determinants of health and the call for an eco-social approach to public health education

**DOI:** 10.17269/s41997-019-00263-8

**Published:** 2019-12-02

**Authors:** Margot W. Parkes, Blake Poland, Sandra Allison, Donald C. Cole, Ian Culbert, Maya K. Gislason, Trevor Hancock, Courtney Howard, Andrew Papadopoulos, Faiza Waheed

**Affiliations:** 1 Ecological Determinants Group on Education, Steering Committee, Ottawa, Canada; 2 School of Health Sciences, University of Northern British Columbia, Prince George, BC Canada; 3 Dalla Lana School of Public Health, University of Toronto, Toronto, ON Canada; 4 Faculty of Medicine, University British Columbia, Vancouver, Canada; 5 Northern Health Authority, Prince George, BC Canada; 6 Canadian Public Health Association, Ottawa, ON Canada; 7 Faculty of Health Sciences, Simon Fraser University, Burnaby, BC Canada; 8 School of Public Health and Social Policy, University of Victoria, Victoria, BC Canada; 9 Canadian Association of Physicians for the Environment, Toronto, Canada; 10 Ontario Veterinary College, University of Guelph, Guelph, ON Canada; 11 Intrinsik, Mississauga, ON Canada

**Keywords:** Ecological determinants of health, Eco-social approaches, Public health education, Professional development, Social determinants of health, Ecosystem approaches to health, Planetary health, Déterminants écologiques de la santé, Approches écosociales, Formation en santé publique, Développement professionnel, Déterminants sociaux de la santé, Approches écosystémiques de la santé, Santé planétaire

## Abstract

As a collective organized to address the education implications of calls for public health engagement on the ecological determinants of health, we, the Ecological Determinants Group on Education (cpha.ca/EDGE), urge the health community to properly understand and address the importance of the ecological determinants of the public’s health, consistent with long-standing calls from many quarters—including Indigenous communities—and as part of an eco-social approach to public health education, research and practice. Educational approaches will determine how well we will be equipped to understand and respond to the rapid changes occurring for the living systems on which all life—including human life—depends. We revisit findings from the Canadian Public Health Association’s discussion paper on ‘*Global Change and Public Health: Addressing the Ecological Determinants of Health*’, and argue that an intentionally eco-social approach to education is needed to better support the health sector’s role in protecting and promoting health, preventing disease and injury, and reducing health inequities. We call for a proactive approach, ensuring that the ecological determinants of health become integral to public health education, practice, policy, and research, as a key part of wider societal shifts required to foster a healthy, just, and ecologically sustainable future.

## Résumé

Notre collectif, appelé Ecological Determinants Group on Education (cpha.ca/EDGE), s’est structuré pour aborder les incidences pédagogiques des appels à la mobilisation de la santé publique autour des déterminants écologiques de la santé; nous exhortons les milieux de la santé à bien comprendre l’importance des déterminants écologiques de la santé publique et à en tenir compte, faisant ainsi écho aux appels de longue date de nombreux acteurs – dont les communautés autochtones – le tout dans le cadre d’une approche écosociale de la formation, de la recherche et de la pratique en santé publique. Les approches pédagogiques détermineront si nous serons en mesure de comprendre les changements rapides qui se produisent dans les systèmes vivants dont dépend toute vie – y compris la vie humaine – et d’y réagir. Nous revenons sur les constatations du document de travail de l’Association canadienne de santé publique intitulé *Les changements globaux et la santé publique : Qu’en est-il des déterminants écologiques de la santé?*, et nous faisons valoir qu’une approche intentionnellement écosociale de la formation est nécessaire pour mieux appuyer le rôle du secteur de la santé dans la protection et la promotion de la santé, la prévention des maladies et des blessures et la réduction des inégalités en santé. Nous réclamons une démarche en amont pour que les déterminants écologiques de la santé fassent partie intégrale de la formation, de la pratique, des politiques et de la recherche en santé publique, car ces déterminants seront un élément essentiel des grands mouvements sociétaux nécessaires pour favoriser un avenir sain, juste et écologiquement durable.

## Introduction

The health implications of far-reaching social and ecological change are creating new demands on how the (public) health sector will prepare for the future. Understanding and responding to the degradation of the living systems on which all life—including the well-being of humans—depends is especially important for public health education. While acknowledging that educational reform is only part of what is needed, we view public health education as a key driver of future practice, policy, and research, and therefore, an important lever of systems change. We argue that a particular type of educational and systemic change is demanded when, consistent with other core public health values, the health community recognizes the deep, reciprocal interconnections among the ecological, social, cultural, and economic factors that determine the health of all who share this planetary home (Benatar and Poland 2015; Canadian Public Health Association (CPHA) 2015; Horwitz and Parkes 2019).

The interacting components of our shared home include social systems tightly coupled with the ecosystems that provide the basis for Earth’s life-supporting systems (Canadian Public Health Association (CPHA) 2015; Hancock et al. 2015; Parkes and Horwitz 2016). The dynamic relationships within ecosystems create the foundations for all life, providing oxygen, water, food, fuels, materials, waste decomposition and recycling, as well as the constituent parts for all life-giving systems necessary for the health of humans and other species (Hancock et al. 2015; Parkes and Horwitz 2016; Whitmee et al. 2015). Consistent with long-standing and ongoing calls from Indigenous communities and knowledge systems (Greenwood et al. 2015; Ratima et al. 2019), closer attention to relationships and interconnections helps to overcome false dichotomies between the ecological and social influences on health, and provides the foundation for what is described here as an eco-social approach.

The descriptor ‘eco-social’ is intentional: focusing attention on the reciprocity among the ecological and the social as essential features of a proactive orientation to future health and collective well-being, especially in the face of rapid planetary-scale ecological changes that threaten human well-being and societal stability (Canadian Public Health Association (CPHA) 2015; Hancock 2015; Hancock et al. 2015; Whitmee et al. 2015). Here, we underscore the particular role of education in drawing attention to the ecological determinants of health (EDoH), as a necessary complement to the social determinants of health (SDoH), and as part of an integrative, eco-social approach to public health. Eco-social education and learning are seen as key contributions within a wider suite of deep reforms to policy and practice required to take decisive and much needed action on extensively documented health, (in)equity, and sustainability challenges (Benatar and Poland 2015; Buse et al. 2018; Canadian Public Health Association (CPHA) 2015; EDGE 2018; Greenwood et al. 2015; Hancock 2015; Hancock et al. 2015; Horwitz and Parkes 2019; Parkes and Horwitz 2016; Poland et al. 2011; Ratima et al. 2019; Rayner and Lang 2012; Webb et al. 2010; Whitmee et al. 2015).

Contributing to the transition to a sustainable, just, and healthy future has become an integral part of the health sector’s role—and responsibility. This need was revisited by the Canadian Public Health Association at the 20th anniversary of the landmark 1992 report on human and ecosystem health and resulted in a discussion paper ‘*Global Change and Public Health: Addressing the Ecological Determinants of Health*’ (Canadian Public Health Association (CPHA) 2015). The CPHA Working Group that contributed to the 2015 discussion paper was informed by its 400+-page ‘full’ report and a 100-page ‘report in brief’ (Hancock 2015). This work addressed what is known about the EDoH, identified key drivers of ecological change, assessed existing and projected population health impacts, laid out the potential for a change of course, and proposed an ambitious agenda for an effective public health response (see Box 1).

**Table:** 

The CPHA report on ‘*Global Change and Public Health: Addressing the Ecological Determinants of Health*’ (Canadian Public Health Association (CPHA) 2015) identified a range of roles and responsibilities for responding to EDoH: (a) Understanding and reporting on the health implications and impacts of our current unsustainable forms of development; (b) Undertaking research into the health implications of ecological change and the health benefits of alternative approaches; (c) Proposing healthier public policies and private and community sector actions that support the transition; (d) Communicating effectively with key stakeholders (including the rest of the health care system and the general public) the importance of this issue, the health implications of our present path, and the health benefits of the transition we require; and (e) Making public health an ally, at all levels, with those working to bring about the transition to a sustainable, just, and healthy future, recognizing that in many cases these partners have decades of experience to draw upon. Calls for educational reform include: • Update Canada’s set of core competencies for public health to give greater prominence to the ecological determinants of health, ensuring that public health practitioners have the ability to address both the ecological and social determinants of health; • Revise the curricula in Canada’s schools and programs of public health to reflect a broader understanding of population health and its determinants, incorporating core concepts or courses that address the ecological determinants of health and links with social determinants; • Encourage awareness of combined approaches to ecological and social determinants of health that will align public health with a range of existing movements spanning environmental, Indigenous, conservation, labour, social justice, climate change efforts, etc.; and • Include learning of a wide range of change-oriented practices employed by diverse actors involved in complexity science, community organizing, social practice theory, interdisciplinary work on governing societal transitions, transformative learning, Theory U, generative dialogue, etc.	

Calls for educational reform were central to the recommendations of the 2015 CPHA report, recognizing the need for a concerted response to meet the education, training, and professional development needs of current and future health professionals to more fulsomely address these emerging challenges (Box 1). In this context, the EDoH can no longer be an educational ‘option’, but rather a foundational component of learning about health: recognized as integral to achieving core public health functions of protecting and promoting health, preventing disease and injury, and reducing health inequities. Recognizing this need, the Ecological Determinants Group on Education (EDGE) was co-created with a range of partner agencies, including CPHA (https://www.cpha.ca/EDGE). EDGE brings together public health and allied professionals, researchers, and educators with interest and expertise in the ecological determinants of health, with the goal of promoting the integration of EDoH within public health education, training, and professional development, attending to issues of content as well as issues of process and pedagogy (including alignments between these), with the intention of supporting and informing practice, research, advocacy, and policy relating to EDoH.

Recognizing the scope of public health education, an initial focus for EDGE involved forming three working groups, focused on graduate curriculum development, continuing professional development for those working in public health, and training/education for health care professionals. Building on insights across these working groups, EDGE undertook a preliminary scan of needs, challenges, and assets in relation to EDoH in the context of public health education in Canada (EDGE 2018). This scan provides an overview of the emerging state of the field, recognizing the connections with converging and inter-related developments relevant to public health, including but not limited to ecological public health (Rayner and Lang 2012), ecohealth, ecosystem approaches to health (Horwitz and Parkes 2019; Parkes and Horwitz 2016; Webb et al. 2010), and the emergence of planetary health (Ratima et al. 2019; Whitmee et al. 2015) among others (Buse et al. 2018). The EDGE report offers an initial appraisal of key educational resources and emerging initiatives (including a matrix of options for integrating EDoH into education strategies: EDGE 2015, Table 1) and highlights overlaps with other timely critiques of the need for the redesign of public health education and practice to respond to contemporary challenges (see Poland et al. 2019; Walpole et al. 2019; Yassi et al. 2017).

The key premises that undergird our work on the EDoH have become clearer over time and are presented here given their relevance to public health education, training, and professional development.

First, we recognize that integrative approaches to health that address both social and ecological determinants of health are not new. There are a range of ongoing efforts to connect equity, ecosystems, and public health that can be and have been applied in practical, clinical, and policy contexts (see, for example, Buse et al. 2018; O’Connell 2017; Parkes et al. 2017; Poland and Dooris 2010; Walpole et al. 2019; Webb et al. 2010; Yassi et al. 2017).

Second, we emphasize that the EDoH are not the same as the field of environmental health and are also not adequately captured within traditional framings of ‘environmental’ determinants of health. While environmental and ecological approaches are complementary, EDoH responds to the need for explicit attention to ecologies and ecosystems as foundational to human health (Horwitz and Parkes 2019; Parkes and Horwitz 2016), recognizing the complex and interacting impacts of climate change, biodiversity loss, pollution, ocean acidification and depletion, land and water degradation, and food security, from local to global scales, as they affect personal, public, and planetary health (Canadian Public Health Association (CPHA) 2015; Greenwood et al. 2015). This emphasis on systems relationships is complementary to, but not the same as, current framings of environmental public health, with its focus on the built environment, food handling, water safety, waste disposal, workplace health and safety, communicable disease control, and more generally the identification and management of known environmental health risks and exposures.

More to the point, occupational and environmental health are, in the main, predicated on a risk management approach that emphasizes the prediction and control of putative ‘threats’ to human health, and privilege efforts to bounce back from adversity in ways that typically maintain the status quo, often placing human health considerations ahead of ecological concerns. While these efforts remain essential, they do not respond to the imperative for transformational change, in the ways that an eco-social approach can: drawing from a more relational world-view, complexity science, and an understanding that human and non-human well-being are inextricably interwoven (Greenwood et al. 2015; Horwitz and Parkes 2019; Poland and Dooris 2010; Ratima et al. 2019; Webb et al. 2010). Resilience in the face of mounting uncertainty and accelerating change involves more than the capacity to bounce back, instead valuing the capacity to embrace change and bounce forward into new ways of seeing and doing (what some call transformative resilience, or transilience), drawing on change processes that are deeply collaborative and emergent, rather than command and control (see O’Connell 2017; Parkes et al. 2017; Poland et al. 2011, 2019).

Third, in keeping with the far-reaching implications of a relational systems orientation, we argue that (public) health efforts to address the EDoH need to acknowledge, engage, and be more actively informed by other holistic and integrative approaches to health and wellness, especially in terms of established leadership of Indigenous peoples and perspectives in integrative understandings of ecological, social, cultural, and intergenerational determinants of health (Buse et al. 2018; Greenwood et al. 2015; Ratima et al. 2019; Rayner and Lang 2012). This fuels the imperative for the health community to commit to an inclusive approach that respects diverse knowledge systems and ways of knowing, and to the challenging but essential work of truth and (re)conciliation that such understandings and inclusions imply, in the spirit of informed allyship and decolonization (O’Connell 2017).

Fourth, we recognize that attention to EDoH and an eco-social approach requires collaboration within and beyond the health sector, creating benefits and challenges across converging efforts, in a space rapidly populated by a variety of overlapping approaches, perspectives, and agendas (Buse et al. 2018; Poland and Dooris 2010). Within the health sector, the EDoH and related eco-social approaches touch on multiple dimensions: crossing boundaries between health protection, health promotion, and health care in ways that may be challenging for traditional roles and responsibilities, and the operational structure of public and population health programs (e.g., where does climate change ‘fit’ within existing public health programming?). Ongoing social and ecological changes also mean that health sector agendas are converging with those of groups and agencies beyond the health sector in new ways. This means working with professional sectors (planning, land and water management, emergency preparedness, etc.); with non-health ministries and municipal governments; with ‘unusual allies’ in civic society (e.g., environmental organizations, social justice movements, Indigenous, and other groups); and, where appropriate, with emerging green/sustainable and social enterprise entrepreneurs in the private sector. While health professionals can provide important perspective and leadership, we also need to support experienced partners from disciplines and fields beyond the health sector that, in many cases, will appropriately continue to lead in the wider community and society, toward the healthy, just, and sustainable future to which we aspire. Health professionals will be called on to engage as humble, informed, and trusted partners in the collective, boundary-crossing effort of transforming practices and structures to better sustain the health and well-being of all life, including our own.

Fifth, we recognize that educational reform, while necessary for proactive change, is not sufficient in and of itself. The systemic drivers of ecosystem degradation and social inequity are deeply entrenched and not easily amenable to change through the application of ‘enlightened’ information alone: the deficit model of educational reform and change-making has underperformed relative to current demands (Parkes et al. 2017; Walpole et al. 2019; Yassi et al. 2017). While EDGE and this commentary have focused on the education implications of embracing eco-social approaches to the EDoH, these need to be seen as synergistic with the urgent need for a fulsome whole-of-society retooling of the systems and power structures that continue to perpetuate both ecological and social degradation (Poland et al. 2011; Ratima et al. 2019; Whitmee et al. 2015). That the prospects for such change seem daunting and hitherto ‘unrealistic’ should not detract from their necessity, or indeed the likelihood that they will be thrust upon us anyway if we fail to embrace them proactively.

These premises and insights underscore the imperative for proactive and assertive efforts to ensure that the EDoH are embedded in public health education, policy, and practice. Given the challenges of the Anthropocene (Buse et al. 2018; Hancock 2015; Whitmee et al. 2015), this can no longer be considered a ‘nice-to’ but rather a ‘need-to’ that will push public health to bring out its best responsive, proactive, and collaborative tendencies. An eco-social approach to the EDoH, informed by the five premises outlined above and aligned in terms of content (concepts and topics) as well as process (pedagogical approaches suited to learning from the land and living systems), is, in our view, an essential step in unleashing the transformative potential of public health in the coming decade.
